# Efficiency of health resource utilisation in primary-level maternal and child health hospitals in Shanxi Province, China: a bootstrapping data envelopment analysis and truncated regression approach

**DOI:** 10.1186/s12913-020-5032-y

**Published:** 2020-03-06

**Authors:** Tao Zhang, Wei Lu, Hongbing Tao

**Affiliations:** Department of Health Management, School of Medicine and Health Management, Tongji Medical College, Huazhong University of Science and Technology, No.13, Aviation Road, Qiaokou District, Wuhan, Hubei Province China

**Keywords:** China, Data envelopment analysis, Maternal and child health hospital, Technical efficiency, Truncated regression

## Abstract

**Background:**

District- and county-level maternal and child health hospitals (MCHHs) are positioned to provide primary maternal and child healthcare in rural and urban areas of China. Their efficiencies and productivity largely affect the equity and accessibility of maternal and child health care. This study aimed to assess the efficiency of district- and county-level MCHHs in China and identify their associated factors.

**Methods:**

Thirty-three district- and 84 county-level MCHHs were selected from Shanxi Province in 2017. At the first stage, bootstrapping data envelopment analysis (DEA) models were established to calculate the technical efficiency (TE), pure technical efficiency (PTE) and scale efficiency (SE) of district- and county-level hospitals. At the second stage, the estimated efficiency scores were regressed against external and internal hospital environmental factors by using bootstrap truncated regression to identify their determinants.

**Results:**

The average TE, PTE and SE scores for district-level MCHHs were 0.7433, 0.8633 and 0.9335, respectively. All hospitals were found to be weakly efficient, although more than 50% of the hospitals performed with efficient SE (SE scores≥100%). As for county-level MCHHs, their average TE, PTE and SE scores were 0.5483, 0.6081 and 0.9329, respectively. The hospitals with TE and PTE scores less than 0.7 accounted for more than 60%, and no hospital was observed to operate effectively. Truncated regressions suggested that the proportion of health professionals, including doctors, nurses, pharmacists, inspection technician and image technician (district level: *β* = 0.57, 95% CI = 0.30–0.85; county level: *β* = 0.33, 95% CI = 0.15–0.52), and the number of health workers who received job training (district level: *β* = 0.67, 95% CI = 0.26–1.08; county level: *β* = 0.34, 95% CI = 0.14–0.54) had a positive association with efficiency scores. The amount of financial subsidy (*β* = 0.07, 95% CI = 0.05–0.09) was found to be directly proportional to the productive efficiency of the county-level MCHHs.

**Conclusion:**

The operational inefficiency of district- and county-level MCHHs in Shanxi Province is severe and needs to be substantially improved, especially in terms of TE and PTE. Hiring additional medical personnel and ensuring the stability of the workforce should be prioritised. The Chinese government must provide sufficient financial subsidy to compensate for service costs.

## Background

The health status of women and children directly reflect the level of national health and social development [1]. The number of women and children accounts for nearly two-thirds of the whole population in China, and maternal and child health care (MCHC) has always been the focus of government welfare programmes [2]. In the eight Millennium Development Goals (MDGs) advocated by the United Nations, two MDGs are directly related to MCHC: MDG 4 (a two-third reduction in under-5 mortality between 1990 and 2015) and MDG 5 (a three-quarter reduction in maternal mortality ratio between 1990 and 2015) [3]. Although the MDGs have come to the end of their term, a post-2015 agenda comprising 17 Sustainable Development Goals are implemented, in which the health and well-being of women and children are still two important goals [4]. Being a United Nations member, the Chinese government has launched the policy of ‘China women and children development programme’ every 10 years since 2001 with the aim of providing equitable and high-quality maternal and child care to protect their basic health rights [5].

China’s one-child policy was replaced by a universal two-child policy (almost all Chinese people can have their preferred number of children) in 2015. Although several studies have indicated that this new policy is unlikely to cause a large increase in the average birth rate in China, some regions, such as rural areas and small towns, may face an increase in fertility due to an unbalanced development level across the country [6]. Therefore, the service capacity of MCHHs should be improved.

In recent years, the Chinese government has made huge investments in MCHC, especially in terms of the physical facilities and human resources of maternal and child health hospitals (MCHHs). Predictably, these efforts have generated remarkable effects. For example, MDG 4, which aims to reduce child mortality by two-thirds, has been reached in advance of 9 years; MDG 5, which seeks to decrease maternal mortality by three-quarters, has been achieved 1 year ahead of schedule [7–9]. However, unequal and inefficient MCHC remains a persistent issue in China, and an MCH service network is lagging in the entire healthcare system [7].

The administrative divisions in China are divided into four levels: provincial, municipal, county/district and township levels. Accordingly, the MCHC system is composed of different levels of MCHHs. Given that MCHHs are absent at the township level in China, county- and district-level MCHHs are positioned to provide primary healthcare institutions. Although county- and district-level hospitals are classified at the same administrative level, they have different functions. County-level MCHHs are located in rural regions and provide MCH services for rural residents. By contrast, district-level MCHHs are located in urban regions and provide MCH services for urban residents [10]. However, the development of both county- and district-level MCHHs has been lagging due to a serious shortage in health resources, particularly in the number of professional health workers [11]. To address these problems, the Chinese government has allotted sufficient funds into the primary healthcare sector, including MCHHs, since China’s 2009 health system reform [12]. Therefore, whether these resources have been well utilised must be examined.

Previous studies that investigated the productive efficiency of MCHHs in China have provided insightful evidence. For instance, XU Yan et al. (2013) measured and evaluated the productive efficiency of 85 county-level MCHHs in Jiangsu Province and found that 58.8% of the hospitals have a low level of productive efficiency [13]. Xuan Wang et al. suggested that the overall operational efficiency of county-level MCHHs in Guangxi is low and needs to be substantially improved [10]. Other relevant studies found that the inefficiency of MCHHs greatly affects the quality and equality of MCH services [10, 14–16].

To date, existing literature has rarely scrutinised the productive efficiency of primary-level MCHHs after China’s new round of healthcare system reforms and their multi-faceted influencing factors, although studies have closely examined the performance of MCHHs in terms of productive efficiency. The objectives of this study were to measure the productive efficiency of county- and district-level MCHHs and identify their associated factors from the perspectives of internal and external environments. We hope to provide appropriate strategies for the sustainable development of primary-level MCHHs in China.

## Methods

### Study setting

Shanxi Province, located in the central region of China, had a population of approximately 36 million in 2017, andthe female and children populations accounted for 48.1 and 15.5%, respectively. Its per capita gross domestic product (GDP) was 40,557 Chinese Yuan in 2017, ranking it 26th amongst 31 provinces and municipalities in mainland China. A total of 134 provincial-, municipal- and district−/county-level MCHHs provide healthcare services across the entire province.

### Data sources

We adopted a census sampling approach to investigate 117 primary-level MCHHs (33 district-level and 84 county-level MCHHs) in Shanxi Province. The self-administered questionnaire related to the input and output of MCHHs was distributed to managers in each hospital via e-mail, and all questionnaires were returned (response rate: 100%) from June to August 2018. After checking the returned questionnaires for completeness, the data were inputted into the database. In addition, some information about the external hospital environment, such as population and GDP, was extracted from Shanxi Statistical Yearbook in 2017. No patient information was included in this study.

### Statistical analysis

This study applied a two-stage analysis strategy: firstly, a bootstrapping data envelopment analysis (DEA) was used to measure productive efficiency of county- and district-level MCHHs. We then adopted bootstrap truncated regression to explore the factors associated with their productive efficiency.

### Stage one: a bootstrapping DEA

DEA is widely used to examine the productive efficiency of health institutions around the world because it does not require assumptions on functional form and can be used for relative productive efficiency analysis with multiple inputs and outputs [17, 18]. The DEA model comprises the CCR model (production is constant return to scale [CRS], where an increase in the input will result in a proportional increase in the output) and the BCC model (production is variable return to scale [VRS], which means that an increase in the input will result in either an increase or decrease in the output when units are not operating at optimum scale) [18]. Technical efficiency (TE) measured by the CCR model may be altered by scale efficiency (SE) [18]. VRS has two dimensions: increasing returns to scale (IRS), that is, increasing the input of one unit brings over one unit increase in outputs; and decreasing returns to scale (DRS), which indicates one unit increase in inputs will result in below one unit increase in output [19]. The BCC model calculates the pure technical efficiency (PTE) that incorporates the effect of SE [18, 20]:

$$ {TE}_{DEA- CRS}={PTE}_{DEA- VRS}\times SE $$

However, all decision-making unit (DMU) scores in the DEA model decrease in a fluctuating range when influenced by environmental and random factors. Thus, using traditional DEA to measure productive efficiency scores may generate bias [21]. To correct this bias, we introduced a bootstrapping technique by simulating the data-generating process to obtain a new estimate for each simulated sample [22]. The simulated data set is approximately equivalent to the original one, which means that the sampling distributions and standard deviations are close to the original ones. The productive efficiency scores estimated via bootstrapping DEA can produce bias-corrected productive efficiency, thereby resulting in highly accurate productive efficiency scores. Thereafter, Simar and Wilson introduced a smooth bootstrapping procedure to accurately estimate the productive efficiency scores, and it has been applied internationally to measure the relative productive efficiency [23, 24].

Furthermore, the use of an output-orientated DEA, where DMUs are given a fixed quantity of resources (inputs) and asked to maximise output, is appropriate because the input of public hospitals is determined centrally by the Ministry of Health in China; hence, hospital managers have no control over the size of the hospitals they run [25]. The output-oriented DEA using a smooth bootstrapping procedure was adopted and operated on STATA 14.0.

### Stage two: bootstrapping truncated regression

Given that the range of relative productive efficiency scores calculated by the DEA model falls between 0 and 1, a Tobit and truncated regression model has traditionally been used to evaluate the factors affecting the productive efficiency [10, 16]. However, Simar and Wilson argued that the use of a Tobit or truncated regression in a two-stage analysis is inappropriate based on two reasons: firstly, the productive efficiency scores estimated by DEA may be corrected with each other; thus, the results in the error term in these models are serially correlated and a standard inference is not valid. Secondly, in small samples, the explanatory variables used in regression analysis may be associated with the variables used for calculating productive efficiency scores in the DEA, thereby establishing a correlation between the error term and the explanatory variables [26, 27]. To avoid these controversial issues, we adopted bootstrapping truncated regression to explore the factors affecting the productive efficiency of MCHHs and performed it using STATA 14.0 following Simar and Wilson [24].

### Variables

In our questionnaire, the input and output variables and several explanatory variables affecting efficiency were included. During the questionnaire design process, we implemented a two-round expert consultation based on literature review to determine which variables need to be investigated. After revising the questionnaire, we structured the final questionnaire (Additional file 1).

### Input and output variables

Proper selection of input and output variables is crucial to accurately measure the relative productive efficiency of MCHHs. In the initial stage, we considered the number of open beds, the number of health workers, the total expenditure, the number of doctors, the number of nurses, the number of devices over 10,000 Chinese Yuan, the hospital area, the total fixed assets, the total service cost and the cost per visit as the input indicators based on previous studies [10, 13, 28]. The initial output variables selected included total revenue, income from medical services, the number of patient discharges, outpatient and emergency visits, the number of health examinations, average inpatient days and bed occupancy rate [10, 16, 29].

To choose more representative ones from the aforementioned indicators, we firstly performed cluster analysis to address the overlap of the capability of variables in explaining the same portion of the outcomes. A correlation matrix was then extracted to identify and eliminate multicollinearity amongst the variables to help us construct a shortlist of the essential and representative variables. Finally, regression analysis was conducted to identify those inputs with high capability in explaining the variations in the selected outputs [30]. A coefficient of determination value of over 0.5 was considered a benchmark to select the final input and output variables. Table 1 shows the final list of the input and output variables.

**Table 1:** Inputs, outputs and explanatory variables and their explanation

Category	Variable (Type of variable)	Explanation
Inputs	Total expenditure (continuous)	Capital consumption and defray in the process of service provision and other activities, including healthcare, drug and medicine expenditures
	Number of doctors (continuous)	Registered doctors at the end of year, excluding retirees and temporary staff
	Number of nurses (continuous)	Registered nurses at the end of year, excluding retirees and temporary staff
	Number of open beds (continuous)	Actual bed used at the end of the year (not registered beds)
	Number of devices over 10,000 Chinese Yuan (continuous)	Sample hospital that owns medical devices worth more than 10,000 Yuan
Outputs	Total revenue (continuous)	Revenue gained from service provision and other activities, including healthcare revenue, drug and medicine sales and financial subsidies
	Number of patient discharges (continuous)	Number of discharged patients after hospitalisation in sample hospitals at the end of year
	Number of outpatient and emergency visits (continuous)	Number of patients coming for outpatient and emergency diagnostic services in sample hospitals at the end of the year
	Number of health examination (continuous)	Number of health check ups for women and children conducted by the sample hospital, including prenatal examination and postpartum visit
Factors	Population (continuous)	Population in the region where the sample hospital is located
	GDP per capita (continuous)	GDP per capita in the region where the sample hospital is located
	Financial subsidy (continuous)	Amount of financial subsidy from the government that hospitals can receive in a financial year
	Proportion of health professionals (continuous)	As a percentage (%) of all employees in the hospital. Health professionals include doctors, nurses, pharmacists, inspection technicians and image technicians
	Number of health workers who received job training (continuous)	Number of health workers in sample hospitals who received related job training at the end of the year
	Average annual income of staff (continuous)	Average annual income of employees in sample hospitals, including wages, bonuses and subsidies

### Explanatory variables affecting efficiency

According to the literature, two types of factors have been proved to affect the productive efficiency of hospitals: the external and internal environmental factors of hospitals. External factors mainly include catchment population, distance, location (urban/rural), economic status, health insurance and occupancy rate. Hospital staff, educational status, income, average length of stay and hospital scale are frequently selected as the internal factors [10, 14–16, 31].

After reviewing the literature, we determined the candidate variables. We conducted a two-round expert consultation to discuss which explanatory variables should be selected from the candidate variables. Finally, population, GDP per capita in the sample region and financial subsidy from the government in a given financial year were selected as the external environmental factors. Internal environmental factors included the proportion of health professionals in the staff, the number of health workers who received job training and the average annual income of the staff (Table 1).

## Results

### Descriptive statistics of the input, output and explanatory variables

Table 2 describes the mean (SD) for the input, output and explanatory indicators of district- and county-level MCHHs. In general, no remarkable differences were observed in terms of the outputs and inputs amongst these two types of MCHHs, although district-level hospitals had more visits and discharged patients compared with county-level hospitals. With regard to explanatory variables, a considerable difference was observed in terms of population and GDP per capita between the district- and county-level MCHHs. Additionally, the number of health workers who received relevant job training in the district-level hospitals was slightly higher than that in the county-level hospitals.

**Table 2:** Statistical description for input, output and explanatory variables of district- and county-level MCHHs

Category	Variable	District level	County level
		Mean ± SD	Mean ± SD
Inputs	Total expenditure (10,000 RMB)	9942.15 ± 791.14	8650.51 ± 118.19
	Number of doctors	18.64 ± 10.97	15.52 ± 9.86
	Number of nurses	14.18 ± 7.08	12.61 ± 11.55
	Number of open beds	31.81 ± 9.84	27.03 ± 11.23
	Number of devices	45.76 ± 6.51	31.90 ± 11.40
Outputs	Total revenue (10,000 RMB)	11,060.88 ± 954.45	9269.32 ± 1063.23
	Number of patient discharges	2190.44 ± 687.19	990.03 ± 176.97
	Number of outpatient and emergency visits	27,175.58 ± 5561.71	18,558.79 ± 2940.86
	Number of health examination	13,756.32 ± 7145.35	13,087.94 ± 3074.63
Factors	Population	444,967.42 ± 197.25	251,604 ± 132.10
	GDP per capita (RMB)	41,566.27 ± 224.18	26,179.51 ± 145.50
	Financial subsidy (10,000 RMB)	7021.21 ± 454.33	6864.05 ± 767.72
	Proportion of health professionals (%)	42.63 ± 3.71	40.34 ± 3.56
	Number of health workers who received job training	35.24 ± 2.50	26.67 ± 3.74
	Average annual income of staff (RMB)	1313.87 ± 126.74	1106.42 ± 162.56

### Bootstrapping bias-estimated productive efficiency scores

**Fig. 1:**
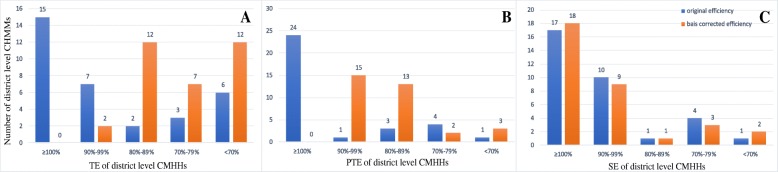
The distribution of TE, PTE and SE in district level MCHHs

**Fig. 2:**
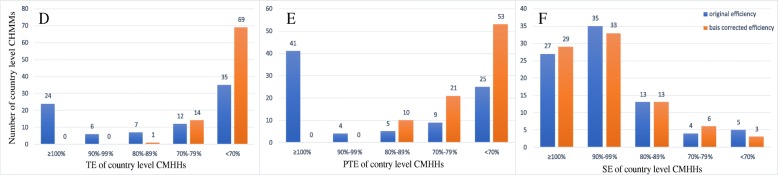
The distribution of TE, PTE and SE in country level MCHHs

Figures 1 and 2 present the distribution of the productive efficiency scores of district- and county-level MCHHs with and without bias corrections. Overall, the productive efficiency scores in the traditional DEA model were higher than those of the bias-corrected scores in bootstrapping DEA.

### Productive efficiency of district-level MCHHs

After correcting bias via a bootstrapping DEA, the mean of TE, PTE and SE in district-level MCHHs was 0.7433, 0.8633 and 0.9335, respectively. This result indicated that these MCHHs should, on average, increase their outputs by 13.67% with the same volume of inputs to achieve an effective level.

Figure 1 shows the distribution of TE, PTE and SE in 33 district-level MCHHs. All hospitals presented a TE and PTE scores less than 1, which meant that inefficiency was serious. Furthermore, the majority of the hospitals had a TE score of less than 0.9, and the hospitals with a PTE score between 0.80 and 0.99 accounted for approximately 85%. For SE, the data indicated that more than a half of MCHHs (54.5%) were operating under CRS, implying that these hospitals had no need to adjust their size to achieve optimal scale.

### Productive efficiency of county-level MCHHs

The productive efficiency of county-level MCHHs was low with average bias-corrected TE and PTE scores of 0.5483 and 0.6081, respectively, whilst the score of SE was 0.9329. If the MCHHs in the sample were operating efficiently, then they should have produced 39.19% more outputs with the same volume of inputs.

As shown in Fig. 2, more than half of the MCHHs garnered TE and PTE scores less than 0.7, suggesting that these hospitals operated at low efficiency. Similar to district-level MCHHs, no hospitals obtained TE and PTE scores equal to 1. SE was less of a problem as the remaining 29 hospitals (34.52%) operated under CRS, and the scores of 33 hospitals (39.28%) ranged from 90 to 99%.

### Difference in productive efficiency between district- and county-level MCHHs

As shown in Table 3, the differences in TE, PTE and SE between the district- and county-level MCHHs were identified via Student’s *t*-test. The TE and PTE scores were significantly different (*p < 0.001*), in which the district-level MCHHs performed more efficiently than the county-level MCHHs. However, the difference was not statistically significant (*p = 0.980*) in terms of SE scores.

**Table 3:** Comparison of difference in TE, PTE and SE scores between district- and county-level MCHHs

	Hospital	N	Mean	S.D.	*P*
TE	District level	33	0.7433	0.1327	< 0.001
	County level	84	0.5483	0.1513	
PTE	District level	33	0.8633	0.0777	< 0.001
	County level	84	0.6081	0.1643	
SE	District level	33	0.9335	0.1350	0.980
	County level	84	0.9329	0.1055	

### Determinants of productive efficiency based on bootstrap truncated regression

The bootstrap truncated regression model considered the TE score as the dependent variable and the external and internal hospital environmental factors as the independent variables. This model was established to analyse the effects of these factors.

As shown in Table 4, the proportion of health professionals (district level: *β* = 0.57, 95% CI = 0.30–0.85; county level: *β* = 0.33, 95% CI = 0.15–0.52) had a positive association with efficiency scores. Similarly, the productive efficiency scores of district- (*β* = 0.67, 95% CI = 0.26–1.08) and county-level MCHHs (*β* = 0.34, 95% CI = 0.14–0.54) increased accordingly with the addition of the number of health workers who received relevant job training. Moreover, the amount of financial subsidies from the government (*β* = 0.07, 95% CI = 0.05–0.09) was proportional to the productive efficiency of county-level MCHHs. With regard to the other factors, the results suggested that they had no significant relationship with the overall productive efficiency (*p* > 0.05).

**Table 4:** Estimation results from bootstrap truncated regressions

Dependent variable	District-level MCHHs		County-level MCHHs	
	*β*	95% CI	*β*	95% CI
Population (in logs)	0.06	(−0.09, 0.21)	0.03	(−0.03, 0.09)
GDP per capita (in logs)	−0.05	(−0.15, 0.03)	−0.03	(−0.09, 0.01)
Financial subsidy (in logs)	−0.02	(−0.25, 0.20)	0.07^**^	(0.05, 0.09)
Health professionals	0.57^**^	(0.30, 0.85)	0.33^**^	(0.15, 0.52)
Health workers who received job training	0.67^*^	(0.26, 1.08)	0.34^*^	(0.14, 0.54)
Average annual income of staff (in logs)	0.02	(−0.18, 0.23)	0.01	(− 0.01, 0.04)
Sigma	0.06		0.05	
Log likelihood	42.69		119.27	
Wald χ2	51.89		512.30	

*: *p* < 0.05; **: *p* < 0.001; 2000 bootstrapping replications were used

## Discussion

This study provides insights into the productive efficiency of district- and county-level MCHHs and their associated factors using the samples from Shanxi Province, China. Overall, these hospitals operated with low productive efficiency, but inefficiency in terms of SE was not obvious.

With respect to district-level MCHHs, all units were found to be inefficient in terms of TE and PTE, and the TE scores for most hospitals (93.9%) were less than 0.9. Thus, the overall technical inefficiency of district-level MCHHs in Shanxi Province is serious but with huge potential for improvement. In other words, the resources invested to MCHHs at their current size are not fully utilised, and their output is insufficient [10, 25]. Similarly, PTE was a widespread problem, that is, the capacity of management and technology in the district-level MCHHs was rather low. Although more than half of the hospitals performed under CRS, the scales of operation of these inefficient hospitals should be adjusted.

The inefficiency of the county-level MCHHs presented in this study was more severe than that of the district-level MCHHs. On the one hand, all hospitals did not achieve an optimal output value, similar to the situation of the district-level MCHHs. On the other hand, an average of 45.17% of the health resources of the county-level MCHHS were wasted, and the majority (80%) performed with a productive efficiency score less than 0.7. These findings suggest that productive efficiency can be improved by optimal utilisation of the available health resources of primary-level MCHHs.

These results were in agreement with those of Athanassopoulos [32], who pointed out that district-level MCHHs have a higher TE and PTE compared with county-level MCHHs. A possible explanation is that the huge disparity in terms of economic status between the rural and urban areas in China results in uneven distribution of health resources, especially in terms of the professional health workforce. Under such a circumstance, district-level MCHHs in urban regions obtain more technical and financial support than county-level MCHHs, allowing the former to operate efficiently [33].

Regression coefficients of the proportion of health professionals and the number of health workers who received job training indicated that these two variables had a significant positive relationship with the overall technical efficiency of district- and county-level MCHHs. These results were consistent with those of other studies. Indeed, health workers, as the core health resource in hospitals, have a direct effect on the quantity and quality of health services and substantially contribute to outputs [11, 34]. However, most skilled health technicians are unwilling to work in primary healthcare sectors due to low income and high-pressure working conditions [6, 7, 10]. These factors explain why these hospitals fail to attract and retain patients, thereby further reducing their productive efficiency. Thus, introducing qualified personnel from the outside and training internal personnel to improve their skills are effective measures to enhance the service capabilities and optimise the outputs of primary-level MCHHs.

With regard to the external environmental factors, the regression model showed a statistically significant and positive relationship between financial subsidy from the government and productive efficiency of county-level MCHHs. As non-profit public hospitals in China, county-level MCHHs provide affordable basic medical services for low-income rural residents and undertake public health projects, such as vaccination for children and postpartum visit, free of charge. The financial subsidy from the government is usually regarded as the main source of hospital income (the proportion of income from financial subsidy to total income, on average, reached 86% in our study). However, in poor counties, MCHHs are unable to receive adequate financial support from their governments. Under such conditions, county-level hospitals lack funds to provide free MCHC, thereby negatively affecting their outputs [35, 36].

Notably, the coefficient of GDP per capital had no significant effect on efficiency scores. This finding was inconsistent with that of other studies [10, 37]. A possible explanation is the huge disparity in the distribution of health resources between primary health institutions and tertiary hospitals. This circumstance forces patients to visit higher level hospitals to receive better quality of services in China [38]. Accordingly, most women and children tend to choose provincial or municipal MCHHs. This tendency may result in a reduced variation in outputs, such as in terms of the number of outpatients and inpatients, amongst developed and underdeveloped areas. Therefore, this variable is not sensitive for productive efficiency in the model.

Several limitations in our study should be mentioned. Firstly, the sample size of 33 district-level MCHHs is relatively small, although we adopted a bootstrapping DEA to correct bias. Secondly, factoring only six independent variables representing external and internal hospital environmental factors is insufficient to explain the variation in productive efficiency scores in bootstrap truncated regressions due to the inaccessibility of other data. Thirdly, given the lack of information about the case mix of each hospital or patient outcome, variables related to service quality (such as risk-adjusted mortality) were not included in the output when we created the DEA model. Therefore, further research should consider the quality of care whenever data are available.

Despite the aforementioned limitations, this study is still considered a laudable attempt to explore the productive efficiency of primary-level MCHHs and identify their associated multi-faceted factors, especially after the implementation of China’s new round of health care system reforms.

## Conclusions

Overall, the efficiency of district- and county-level MCHHs is rather low and needs to be improved, especially in terms of TE and PTE. The factors that are significantly associated with productive efficiency have several implications. Firstly, introducing additional medical personnel and ensuring the stability of the workforce should be prioritised. Secondly, vocational training and continuing education for health care technicians must be provided to enhance their medical skills and improve the efficiency of primary-level MCHHs. Thirdly, the government needs to provide sufficient financial subsidy for primary-level MCHHs to compensate for service costs.

## Supplementary information

**Additional file 1.** Questionnaire.

## Data Availability

The data sets analysed during this study are available from the corresponding author upon reasonable request.

## Abbreviations

CRS: Constant return to scale
DEA: Data envelopment analysis
DMU: Decision-making unit
DRS: Decreasing returns to scale
IRS: Increasing returns to scale
MCHC: Maternal and child health care
MCHHs: Maternal and child health hospitals
MDGs: Millennium Development Goals
PTE: Pure technical efficiency
SE: Scale efficiency
TE: Technical efficiency
VRS: Variable return to scale
