# Supplementary material for: Efficiency of health resource utilisation in primary-level maternal and child health hospitals in Shanxi Province, China: a bootstrapping data envelopment analysis and truncated regression approach
Source: BMC Health Serv Res. 2020 Mar 6;20:179. doi: 10.1186/s12913-020-5032-y (PMC7059375; doi:10.1186/s12913-020-5032-y)
Supplement: Supplementary file 1 — Additional file 1. Questionnaire. [file 12913_2020_5032_MOESM1_ESM.docx]

Questionnaire

1. How many open beds in this hospital in 2017? ________beds
2. How many health workers in this hospitals in 2017? ________persons
3. What is the total expenditure in this hospitals in 2017? _______yuan
4. How many doctors in this hospitals in 2017? _______doctors
5. How many nurses in this hospitals in 2017? _______nurses
6. How many devices over 10000 Chinese Yuan in this hospitals in 2017? _______devices
7. What is the area of this hospital in 2017? _____m^2^
8. What is the fixed assets of this hospital in 2017? _____yuan
9. What is the total services cost of this hospitals in 2017? _______yuan
10. What is the total revenue of this hospitals in 2017? _______yuan
11. What is the income from medical services of this hospitals in 2017?____yuan
12. What is the total number of discharged patients in this hospital in 2017? ____patients
13. What is the total number of outpatient and emergency visits in this hospital in 2017? ______visits
14. What is the total number of the number of health examinations in this hospital in 2017?
15. What is the average length of stay in this hospital in this 2017? ____days
16. What is the bed occupancy rate in this hospitals in 2017? ______%
17. What is the financial subsidy from the government received by this hospital in 2017? _____yuan
18. What is proportion of health professionals in the staff in this hospitals in 2017? ______%
19. What is the number of health workers who received job training in this hospitals in 2017? ____persons

20.What is the average annual income of the staff in this hospitals in 2017? ______yuan
